# Cure for Asthma

**Published:** 1886-03

**Authors:** 


### THE CURE OF ASTHMA.

In a recent communication to the *Medical Record*, Dr. Richard B. Faulkner says: "I understand by the term asthma, the condition of spasm of the bronchial tubes of both lungs, with hyperæmia approaching or amounting to inflammation, accompanied by rales upon both inspiration and expiration, with great difficulty of breathing, and the term is applied to the paroxysm alone, which returns at regular or irregular periods. Disturbance of function or disease of structure of the pneumogastric nerve is always present.

To cure the paroxisms I originated a method of treatment nearly five years ago; and repeated observation has confirmed its great utility. When called to a case of asthma, with a camel's-hair brush I make a streak of Churchill's iodine over each pneumogastric nerve in its course in the neck, from the upper part of the thyroid cartilage to the upper borders of the clavicles. By counter-irritation thus applied, the capricious and abnormal exercise of nerve-force by the pulmonary filaments is controlled, and bronchial spasm promptly relinquished. Such is my original method—simple, certain, quick. Churchill's tincture is the best counter-irritant, because, first, it is convenient; second, its action is easily controlled; third, it does the work. To permanently cure the paroxysms, it is usually necessary to remove the underlying morbid condition upon which they depend or are associated.

